# Constructing an Emotion Estimation Model Based on EEG/HRV Indexes Using Feature Extraction and Feature Selection Algorithms

**DOI:** 10.3390/s21092910

**Published:** 2021-04-21

**Authors:** Kei Suzuki, Tipporn Laohakangvalvit, Ryota Matsubara, Midori Sugaya

**Affiliations:** Shibaura Institute of Technology, Tokyo 135-8548, Japan; ma21072@shibaura-it.ac.jp (K.S.); ryota@sic.shibaura-it.ac.jp (R.M.); doly@shibaura-it.ac.jp (M.S.)

**Keywords:** emotion recognition, electroencephalogram (EEG), photoplethysmography (PPG), machine learning, feature extraction, feature selection

## Abstract

In human emotion estimation using an electroencephalogram (EEG) and heart rate variability (HRV), there are two main issues as far as we know. The first is that measurement devices for physiological signals are expensive and not easy to wear. The second is that unnecessary physiological indexes have not been removed, which is likely to decrease the accuracy of machine learning models. In this study, we used single-channel EEG sensor and photoplethysmography (PPG) sensor, which are inexpensive and easy to wear. We collected data from 25 participants (18 males and 7 females) and used a deep learning algorithm to construct an emotion classification model based on Arousal–Valence space using several feature combinations obtained from physiological indexes selected based on our criteria including our proposed feature selection methods. We then performed accuracy verification, applying a stratified 10-fold cross-validation method to the constructed models. The results showed that model accuracies are as high as 90% to 99% by applying the features selection methods we proposed, which suggests that a small number of physiological indexes, even from inexpensive sensors, can be used to construct an accurate emotion classification model if an appropriate feature selection method is applied. Our research results contribute to the improvement of an emotion classification model with a higher accuracy, less cost, and that is less time consuming, which has the potential to be further applied to various areas of applications.

## 1. Introduction

In recent years, there has been a number of studies on estimating human emotions in the engineering field, and there are a wide variety of fields where this technology is expected to be applied [1,2,3]. In human–robot interactions (HRI), emotion estimation technology is used to facilitate communication between humans and robots in real-life settings, such as schools [4], homes [5], ambient assisted living [6], hospitals [7], and in rehabilitation [8]. In the field of marketing, the best advertisements [9] for a customer are presented by estimating a customer’s emotion. Furthermore, in the field of education, emotion analysis technology is used to improve the learning process and remote teaching [4]. In daily-living scenarios, such as in homes and ambient assisted living, several sensor technologies have been used to recognize emotions, aiming at improving emotional health and comfort, especially for older adults and people with disabilities [5,6]. In the medical field, mental healthcare is done by detecting unpleasant emotions, such as stress [10], and by assisting people who have communication difficulties due to handicaps [11].

Emotion estimation, which is applicable in various fields, can be divided into several methods. We divided them into two categories, based on the literature of Wang et al. [12] and Jianhua et al. [3]. The first is a method that analyzes facial expressions, posture, behavior, voice, etc. The second is a method that analyzes the autonomic nervous system using physiological indexes, such as electrocardiogram (ECG), respiration, heartbeat, electroencephalogram (EEG), electromyography (EMG), and eye movements. The former method is the result of the intervention of cognitive functions that people can express intentionally and has the advantage of being observable from the outside. However, it can be faked; for example, when a person expresses something different from his or her true intentions. This means that we may not be able to guarantee that an emotion can be estimated accurately [13]. The other is a method for estimating emotions based on direct physiological responses to stimuli, unlike faces and voices, and has received a great deal of attention in recent years [14,15]. Since physiological response to an external stimulus is difficult to change arbitrarily via human consciousness, the latter method has the advantage of being able to estimate emotions more objectively using physiological data [3,12,16].

In the early stages of emotion estimation by analyzing physiological data, the use of a single type of physiological index was the main method. For example, Krishna et al. employed EEG signals to classify basic emotions using a mixture classification technique [17]. However, in recent years, it is known that more complex emotion estimations with a high accuracy can be achieved by using multiple sources of physiological indexes [13].

**Figure 1:**
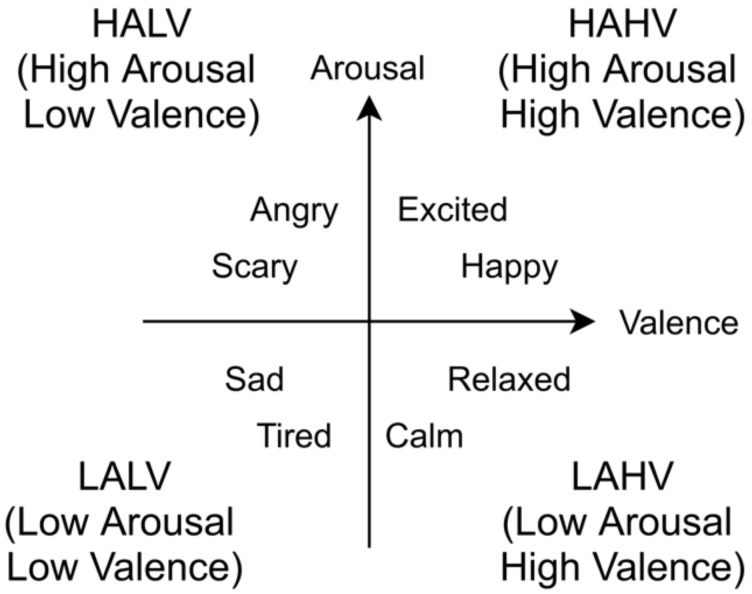
Arousal–Valence space model.

To estimate emotions by analyzing physiological indexes, the Russell’s circumplex model [18] and the Arousal–Valence space model [19] are often used (Figure 1). These models are among the most referenced emotion classification methods in the field of psychology, and represent basic human emotions on two axes, Arousal and Valence [3,14]. This model is also commonly used in studies to estimate emotions by analyzing physiological indexes and is regarded as a proven emotion classification model [2]. Several studies on emotion recognition using multimodal physiological signals have been reported, in which both basic and complex emotion recognition models have been proposed, combining physiological signals, especially EEG, ECG, and EMG [20]. Additionally, anxiety level recognition using blood volume pressure (BVP) and galvanic skin response (GSR), as well as skin temperature, has been proposed recently in areas of application like VR-based therapy [21].

In emotion estimation studies using multimodal physiological indexes, the issue is how to combine physiological and psychological indexes. Ikeda et al. proposed an emotion estimation method that combines EEG and heart rate variability (HRV) indexes with psychological indexes based on Russell’s circumplex model [16]. Ikeda et al. assigned EEG indexes to the Arousal axis of the psychological index (vertical axis of the Russell’s circumplex model), and the HRV indexes to the Valence axis of the psychological index (horizontal axis of the Russell’s circumplex model). Then, the correlation between EEG and HRV indexes were measured in real time and the psychological indexes were analyzed to classify emotions. It has been reported that EEG can be used to measure the state of concentration [22] and that it has a negative correlation with a subjectively evaluated level of arousal [2]. Therefore, we believe that there is a certain validity in mapping EEG indexes to Arousal. The HRV indexes have been considered reliable to detect stress as unpleasant emotions [23]. In addition, some HRV indexes can be used to estimate a relaxed state [2]. Therefore, we believe that there is a certain amount of validity in assigning the HRV indexes to Valence.

However, the method of Ikeda et al. does not take the individual differences that occur in physiological indexes when mapping EEG and HRV indexes to Arousal and Valence, respectively, into account. They mapped pNN50, one HRV index, to Valence. According to Francesco et al., the mean value of pNN50 is 0.3 [24]. In addition, Michael et al. reported that pNN50 is negatively correlated with stress level calculated using a self-assessment questionnaire [25]. Based on the above points, Ikeda et al. employed 0.3 as a threshold of pNN50: emotion is judged as having a high valence if pNN50 is above 0.3 and a low valence if it is below 0.3. However, for more general applications, it is necessary to deal with individual differences in physiological indexes, such as EEG and HRV indexes.

To address this issue, Urabe et al. proposed a machine learning method based on these physiological indexes and the ground-truth information acquired from a self-assessment [26]. They used deep learning with EEG and HRV indexes as features to construct an emotion estimation model for each individual, which enabled highly accurate emotion estimation. As a result of verifying the accuracy of emotion estimation using Urabe et al.’s method, it was reported that an average classification accuracy of 80% and a maximum classification accuracy of 100% were obtained in the four quadrants of the Arousal–Valence space model, HAHV, HALV, LALV, and LAHV, shown in Figure 1 [25].

However, when considering applications in medical fields, such as healthcare, an average accuracy of 80% may still be insufficient. In general, when constructing an estimation model using machine learning, it is suggested that the accuracy can be improved by discovering useful features for estimation and removing unnecessary ones through the calculation, extraction, and selection of features [27]. However, the number of features used in Urabe et al.’s study was only six; five for EEG indexes and one for HRV index, which supports the idea that that the lack of feature extraction and selection is one of the reasons for insufficient accuracy of their emotion estimation model.

Another study that used EEG and HRV indexes as features is that of Tong et al. [28]. They used a total of 34 physiological indexes: 9 HRV indexes from a photoplethysmogram (PPG) data and 25 EEG indexes from a five-channel EEG data. They reported that the machine learning accuracy using all indexes as features was 67% for binary classification of low and high arousal, and 65% for binary classification of low and high valence. However, they did not perform feature selection. By selecting features, we expected a higher accuracy in emotion estimation.

On the other hand, Katsigiannis et al. extracted 42 EEG-based features from 14-channel EEG data and 71 HRV-based features from ECG data [13]. They reported that the accuracy of emotion estimation using all of these features for the binary classification of both high/low arousal, as well as high/low valence, was about 62%.

Katsigiannis et al. extracted a larger number of features than Urabe et al. and Tong et al. However, feature selection was still not performed. In addition, there is an issue with the measurement equipment used for feature extraction. When wearing an EEG sensor, the electrodes need to touch the scalp, and hair needs to be avoided. In addition, some EEG sensors require saline solution or special gel to reduce the electrical resistance. To increase user comfort and ease of use, it is recommended to use fewer electrodes [14], but Katsigiannis et al. used 14 electrodes. In addition, although they used an ECG to calculate the HRV indexes, a PPG can be an alternative to measure the same indexes more inexpensively [2].

Therefore, our study used a simple single-electrode EEG sensor to increase user comfort and ease of use, and a PPG sensor to collect HRV data at a low cost, in order to verify whether emotion estimation technology can be performed more easily. In addition, we extracted and selected the features from EEG and HRV data, aiming at increasing the accuracy of emotion estimation model. In this paper, our method for feature extraction and selection, construction of deep-learning-based emotion estimation model, and validation of the model accuracy are presented.

The structure of this paper is as follows: Section 2 describes the EEG and HRV indexes to be used as features for model construction via machine learning; Section 3 describes the data collection method for model construction; Section 4 describes our proposed method, that is, the feature selection and its results; Section 5 describes the proposed emotion classification model and its accuracy validation results; and Section 6 summarizes the paper.

## 2. Feature Extraction from EEG/HRV Data

### 2.1. EEG Indexes

EEG is an electrical signal recorded in the brain using electrodes. The EEG signal is classified into several bands based on the frequency, each of which has different interpretations in psychological and brain activity states [29]. Generally, wide frequency bands such as α, β, and γ, are used as indexes to estimate human emotions. In addition, subdivided frequency bands such as low α and high α can be used to estimated more subtle human emotions. Therefore, we employed all of them as EEG indexes in this study.

In addition to the above EEG indexes, we used moving average of those EEG indexes with a window size of 15. Since EEG indexes have severe fluctuations by nature, which is considered to inhibit the effectiveness of the objective function minimization, and the threshold value calculation of the machine learning algorithm, calculating the moving average is considered to help reduce this inhibition, and may result in the increase of accuracy of emotion estimation model. Table 1 shows the EEG indexes and their corresponding frequency bands and interpretations used in this study [30,31,32].

Urabe et al. evaluated the function of the frontal lobe in brain function in order to measure Arousal in the Arousal–Valence space model [25]. EEG signal acquired from the frontal lobe is often used for an integrated measurement of concentration and drowsiness. In addition, some studies reported that emotion estimation accuracy of 90% or more could be achieved only with a couple of electrodes placed in the frontal lobe [3]. Therefore, this study also measured EEG signals by placing an electrode near the left frontal lobe, namely the AF3 channel as defined by the International 10–20 EEG system, using Mindwave Mobile 2 manufactured by NeuroSky as EEG sensor, which is a simple and low-invasive single-channel EEG sensor with a sampling rate of 512 Hz. The output from this EEG sensor is acquired approximately once per second.

Although the EEG indexes α, β, and γ cannot be acquired directly from this EEG sensor, they can be calculated from the raw data: Low α + High α for α; Low β + High β for β; and Low γ + Mid γ for γ. Note that raw data acquired from this sensor represent the relative EEG powers calculated using NeuroSky’s original algorithm and therefore has no units [33].

### 2.2. HRV Indexes

HRV is the physiological phenomenon of the variation in the time interval between adjacent heartbeats or inter-beat interval (IBI). We used pulse wave sensor manufactured by Switch Science that works with Arduino kit to acquire PPG signal. It has the sampling rate of 500 Hz and gives output approximately once per 0.5–1.5 s.

To extract HRV indexes, we employed two most widely used methods: time-domain and frequency-domain. Table 2 shows the HRV indexes and the corresponding interpretation employed in this study [34,35].

**Figure 2:**
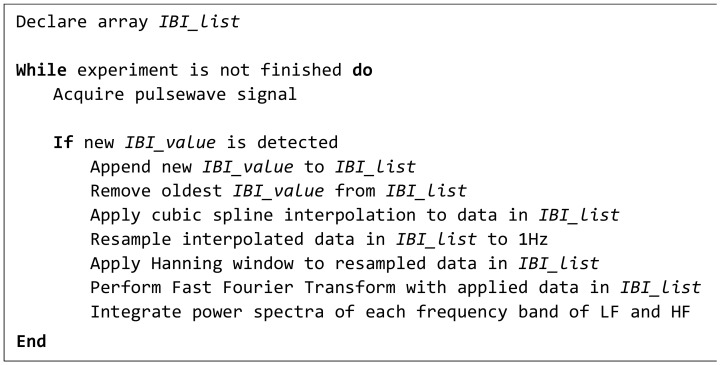
Pseudocode showing the calculation method of LF and HF.

HRV indexes are reported to be influenced by the sympathetic and parasympathetic nervous systems. LF and HF, which are frequency-domain HRV indexes, are decomposed from pulse wave signal into high-frequency (HF) and low-frequency (LF) domains using fast Fourier transform (FFT). LF is considered to the reflect sympathetic nerve, and HF is considered to reflect both parasympathetic and sympathetic nerves. Human emotions can be evaluated by using the ratio of LF and HF (LF/HF) [35]. We described the calculation method of LF and HF as a pseudocode, shown in Figure 2.

In addition to LF and HF as frequency-domain HRV indexes, the standard deviation and coefficient of IBI variations can be used as time-domain HRV indexes for sympathetic and parasympathetic nerves. We calculated several indexes (i.e., pNNx, SDNN, RMSSD, SDNN/RMSSD, and CVNN) using 30 IBI datapoints as a sliding window size, meaning that these indexes are newly calculated each time the new IBI is acquired.

## 3. Data Collection

To prepare datasets for constructing emotion estimation model by machine learning, we needed to collect both EEG and HRV data and label them with corresponding emotions. This section describes an experimental method used to acquire these data. In addition, we describe how to prepare a dataset for machine learning using the collected data. The participants in this experiment were 25 adults who were in their 20s (16 Males; 6 Females) and 30s (2 Males; 1 Female). All of them are Japanese and were physically and emotionally healthy.

### 3.1. Emotional Stimulus

In this study, we used music as emotional stimulus employed from a music database created by researchers at Jyväskylä University under repeated consultation with professional musicians [30]. This database contains 110 film soundtracks, each of which is approximately 15 s long. All music was scored by professional musicians based on the dimensional and discrete emotion model into several emotions, such as valence, energy, tension, anger, fear, happy, sad, beauty, etc. For each quadrant of the Arousal–Valence space model, we selected two songs based on highest scores of corresponding emotions as follows: In HAHV, we used songs No.23 and No.24, which have the highest energy scores. In HALV, we used songs No.11 and No.68, which have the highest fear scores. In LALV, we used songs No.33 and No.109, which have the highest sad scores. In LAHV, we used songs No.41 and No.42, which have the highest beauty or tenderness scores (refer to [30] for a full details of the music database and their scores).

### 3.2. Emotion Estimation toward Stimulus

**Figure 3:**
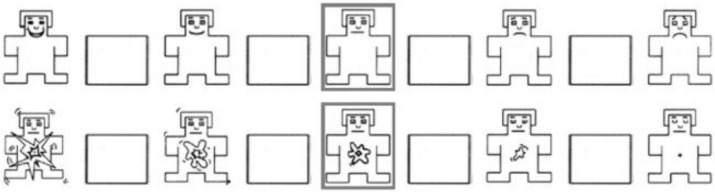
Self-Assessment Manikin (SAM) used for estimating emotions toward music stimuli. The upper scale is for the evaluation of the Valence level. The lower scale is for that of the Arousal level.

We performed subjective evaluation to estimate emotions as arousal and valence towards the eight selected songs using Self-Assessment Manikin (SAM). It is a non-verbal emotion evaluation method that can be performed by selecting one of nine mannequins that most closely resembles one’s emotions (Figure 3). As SAM can be performed regardless of language, we expected that the influence by an individual difference on how one perceives the word can be reduced [36]. In this experiment, as some participants might not be accustomed to self-assessment of emotions using SAM; we asked them to practice using a simple experiment before starting the real experiment.

From the results of SAM, we determined emotion corresponding to each song based on the Arousal–Valence space model. A threshold of 5 was used as it is the mid-point on the SAM scale of 1 to 9, that is, an emotion evaluated with valence ≥5 is judged as high valence and vice versa. Similarly, an emotion evaluated with arousal ≥5 is judged as high arousal and vice versa. Based on these criteria, we divided the evaluated emotion-based Arousal–Valence space model into four classes, as follows:

- Emotions with Arousal > 5 and Valence ≥ 5 or Arousal = Valence = 5 belong to HAHV (the first quadrant);
- Emotions with Arousal ≤ 5 and Valence > 5 belong to HALV (the second quadrant);
- Emotions with Arousal < 5 and Valence ≤ 5 belong to LALV (the third quadrant);
- Emotions with Arousal ≥ 5 and Valence < 5 belong to LAHV (the fourth quadrant).

Based on the above thresholds, we generated categorical data by dividing them into four classes (i.e., HAHV, HALV, LALV, and LAHV): two classes of valence (low/high valence) and two classes of arousal (low/high arousal).

### 3.3. Experimental Procedure and Environment

**Figure 4:**
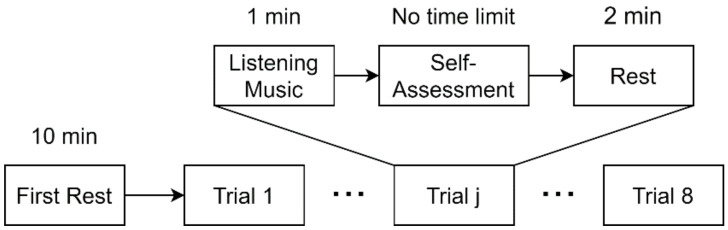
Experimental procedure.

The experimental procedure (Figure 4) is described as follows:

1. Participant sits on a chair and wears EEG sensor, pulse sensor, and earphone. Then, the recording of EEG and pulse wave data is started.
2. Participant practices the experiment by using simplified procedures of steps (3) to (4).
3. Participant waits for 10 min in a resting state (The first rest).
4. Participant listens to the music for 1 min (the same 15-s song is repeated 4 times) and then uses SAM to perform self-assessment of his/her emotion evoked by the music with no time limit. Then, he/she rests for 2 min.
5. Steps (3) and (4) are repeated until eight trials are finished. (Note that the music is changed for each trial). Then, the recording of EEG and pulse wave data is stopped.

In resting and music listening states, an image with a gray background and a black cross in the center was shown on the display placed in front of the participants. Participants were instructed to focus on the cross as much as possible in order to reduce unintentional visual noise. In addition, the experiment was conducted in a quiet room while the participants were wearing earphone at all time in order to reduce unintentional audio noise.

### 3.4. Dataset Construction

From the experiment, we determined sections (start/stop timestamps) when each music stimulus was presented in order to collect EEG and pulse wave data to calculate the EEG and HRV indexes. Since EEG and pulse wave sensors were unsynchronized, the data were fetched from the most recent EEG and pulse wave data, every second. Since each of the music stimulus was presented for about 60 s, approximate 60 EEGs and pulse wave data were generated for each. Subjective evaluation results were also assigned to the physiological data of the corresponding music. These steps were repeated eight times for eight selected songs. Finally, we constructed a dataset as input for machine-learning-based classification models using EEG and HRV indexes as input features and three types of classified emotions from Arousal and Valence scores as emotion labels of the input features. These three types were used for three types of emotion classification models: (1) four-class model for “HAHV/HALV/LALV/LAHV”, (2) binary model for “Low arousal/High arousal”, and (3) binary model for “Low valence/High valence”.

**Figure 5:**
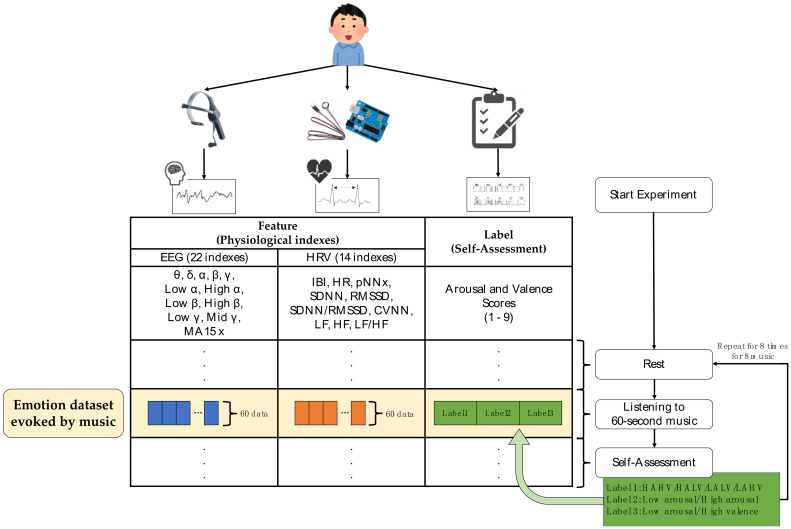
The illustration of the procedure to construct emotion dataset using EEG and HRV indexes as input features for machine-learning-based classification models and three types of classified emotions from self-assessment scores (Arousal and Valence) as three types of emotion labels.

As a result, the constructed dataset contained 3558, 2175, 2704, and 3312 datapoints for the four quadrants in the Arousal–Valence space model. The procedure of dataset construction is illustrated in Figure 5.

## 4. Feature Selection

In this research, we proposed feature selection as a method to improve model accuracy, which was not employed in the research of Urabe et al. [26]. To select the features, we used the degree of contribution technique to generate the feature importance used for feature ranking from multiple feature selection algorithms; an ensemble approach. It was verified by Haq et al. that the ensemble feature selection method yielded a higher accuracy for emotion estimation compared with a single algorithm [37].

We used the following four feature selection algorithms: correlation ratio (CR), mutual information (MI), importance of random forest (RF), and weight of SVM L1 regularization (SVM L1). They were employed for two reasons: (1) they have already been proven, and (2) the feature importance can be calculated to make feature selection easier [38,39,40]. Each feature selection algorithm and the procedure to combine their results are described in the following sections.

### 4.1. Correlation Ratio (CR)

The correlation ratio is a value that quantifies the relationship between qualitative data and quantitative data. Categorical data from the SAM results were used to identify which quadrant in the Arousal–Valence space model the emotion belongs to. Correlation ratios were used to observe the relationship between the emotions in the four quadrants as qualitative data and the EEG/HRV indexes as quantitative data.

The calculation method is expressed by Equation (1). The definitions of the variables in the formula are as follows: $\eta^{2}$ denotes the correlation ratio; a denotes number of qualitative data types; $n_{i}$ denotes number of features x data belonging to the i-th qualitative data; $\bar{x_{i}}$ denotes mean value of features x belonging to the i-th qualitative data; $\bar{x}$ denotes mean value of feature x; and $x_{i,j}$ denotes the value of the  j-th feature x belonging to the i-th qualitative data.

(1) $$\eta^{2}=\frac{\sum_{i=1}^{a}n_{i}{\left(\bar{x_{i}}-\bar{x}\right)}^{2}}{\sum_{i=1}^{a}\sum_{j=1}^{n_{i}}\left(x_{i,j}-\bar{x}\right)}$$

### 4.2. Mutual Information (MI)

Mutual information is a quantified value of the relationship between two variables. In this study, we quantified the relationship between the emotions in the four quadrants and the EEG/HRV indexes.

The formula for calculating the amount of mutual information between qualitative data and quantitative data is as shown in Equation (2) [39]. The definitions of the variables in the formula are as follows: I(X; Y) denotes mutual information of X and Y; p(x) denotes probability of x; p(y) denotes probability of y; and p(x, y) denotes conditional probabilities of x and y.

(2) $$I\left(X;Y\right)=\underset{x,y}{\sum }p\left(x,\;y\right)\;\log\left(\frac{p\left(x,y\right)}{p\left(x\right)p\left(y\right)}\right)$$

### 4.3. Importance of Random Forest (RF)

The importance of a random forest is a quantified value of the degree of contribution in estimating each feature, which is calculated by a machine learning algorithm. Random forest creates multiple decision trees, and the data are classified at the nodes in each decision tree. It is an algorithm that makes a final estimation by voting the classification results based on those decision trees.

The calculation method is expressed by Equations (3) and (4). The definitions of the variables in the formula is as follows: $I_{x}$ denotes importance of feature x; N denotes number of nodes branched by feature x; ${\Delta I}_{x,n}$ denotes the amount of decrease in purity at the nth node branched by the feature x; $G_{\mathrm{Parent}}$ denotes impurity in the parent node of the nth node; $G_{\mathrm{Child}\;\mathrm{Left}}$ denotes impurity in the left child node in the nth node; $G_{\mathrm{Chid}\;\mathrm{Right}}$ denotes impurity in the right child node in the nth node; m denotes number of data in the nth node; $m_{\mathrm{Left}}$ denotes number of data in the left child node in the nth node; and $m_{\mathrm{Right}}$ denotes number of data of the right child node in the nth node.

(3) $$I_{x}=\overset{N}{\underset{n=1}{\sum }}{\Delta I}_{x,n}$$

(4) $${\Delta I}_{x,n}=G_{\mathrm{Parent}}-\frac{m_{\mathrm{Left}}}{m}{\times G}_{\mathrm{Child}\;\mathrm{Left}}-\frac{m_{\mathrm{Right}}}{m}{\;\times G}_{\mathrm{Chid}\;\mathrm{Right}}$$

At each node in multiple decision trees, the amount of decrease in impurity is calculated by classifying the ground-truth data as in Equation (3). The decrease in the impurity can be interpreted as an increase in the purity, which contributes to the classification and estimation. Therefore, the degree of contribution in estimation is quantified by taking the sum, as shown in Equation (4).

For the implementation of the random forest algorithm, we employed Scikit-learn Python-based machine-learning library. The parameter settings are as listed below.

- The number of trees in the forest: 1000
- Criterion: Gini impurity (default)
- The maximum depth of the tree: None (default)
- The minimum number of samples required to split an internal node: 2 (default)
- Bootstrap: True (default)
- All other required parameters are set as default by the library.

### 4.4. SVM L1 Regularization Weight (SVM L1)

Support vector machine (SVM) L1 regularization weight is the weight vector for each feature when the L1-norm regularization term is introduced into the SVM objective function. By introducing the regularization term, the weighting coefficients of the features that are not useful for estimation approach zero, and their influences are reduced, and thus the estimation accuracy is improved [41]. At this time, feature selection was performed by removing the features whose weighting coefficient was close to zero, considering as features not useful for estimation. The equation in which the L1-norm regularization term is introduced into the objective function of SVM is shown in Equation (5) [39]. The meaning of the variables in the formula is as follows:$\;{\left|\left|w\right|\right|}_{1}$ denotes L1-norm term; w denotes weight coefficient; and C denotes variable that controls the degree of influence of the L1-norm term.

(5) $$\underset{w_{0},w}{\min}\overset{n}{\underset{i=1}{\sum }}[1-y_{i}(w_{0}+\overset{q}{\underset{j=1}{\sum }}w_{j}x_{i,J})]+C{\left|\left|w\right|\right|}_{1}$$

For the implementation of the SVM algorithm, we employed Scikit-learn Python-based machine-learning library. The parameter settings are as listed below.

- Kernel: Linear
- The norm used in the penalization: L1 (assigning coefficients/weights to the features)
- Regularization parameter (C): 1.0 (default)
- All other required parameters are set as default by the library.

### 4.5. Feature Selection Ensemble

**Figure 6:**
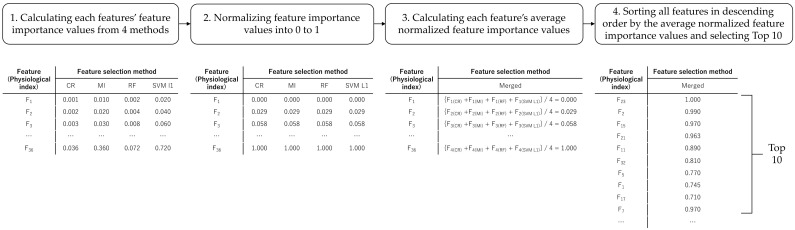
The illustration of the procedure of feature selection ensemble from the calculation of feature importance values by integrating the four feature selection methods to the selection of top 10 important features.

To integrate the results from multiple feature selection algorithms, we performed the following steps (Figure 6):

1. The feature importance of each feature was calculated for each feature selection algorithm. Note that the features are the physiological indexes consisting of 22 EEG indexes and 14 HRV indexes.
2. The feature importance values were normalized so that the maximum value was 1 and the minimum value was 0.
3. For each feature, the average normalized feature importance values were calculated from the values of the four feature selection algorithms.
4. All features were sorted in descending order by the average normalized feature importance values.
5. The indexes in the top 10 were selected as important features.

**Figure 7:**
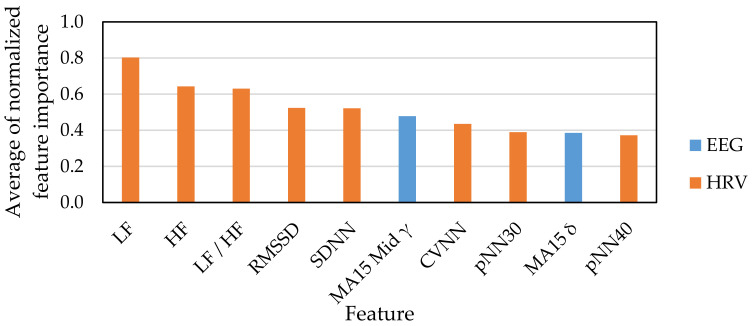
Feature selection result for HAHV, HALV, LALV and LAHV.

**Figure 8:**
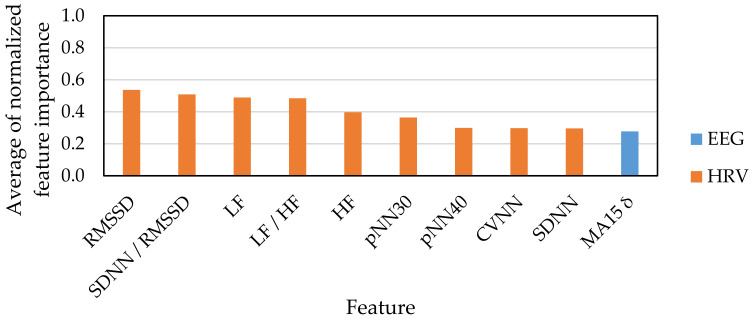
Feature selection result for low arousal and high arousal.

**Figure 9:**
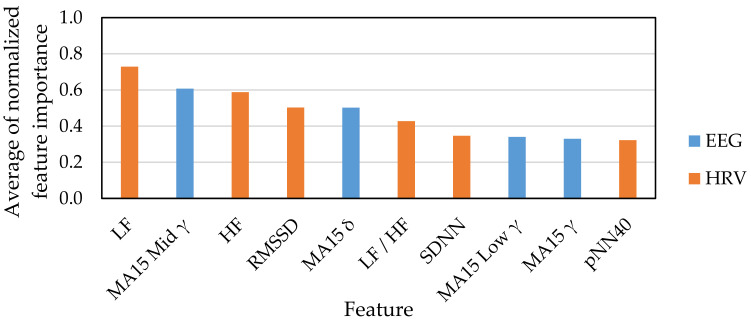
Feature selection result for low valence and high valence.

Figure 7 and Figure 8 show the top 10 indexes that were judged as useful features for emotion estimation based on feature selection results. Figure 7 shows the result of feature selection for the classification of emotions into four classes: HAHV, HALV, LALV and LAHV. Figure 8 shows the classification of emotions into two classes: low arousal and high arousal. Figure 9 shows the classification of emotions into two classes: low valence and high valence.

For EEG indexes, the feature selection results of the four emotion classifications of HAHV, HALV, LALV, and LAHV (Figure 7) suggest that the moving average of γ, that is the index acquired from the high-frequency-band EEG signal, relatively contributes to emotion estimation. This is consistent with the results reported by Wang et al. using a multi-channel EEG sensor [12] and that the γ frequency band is the most sensitive to emotional changes [3]. However, since the EEG signals were acquired only from the frontal lobe (AF4) in this study, it is suggested that the moving average of δ, that is the index acquired from the low-frequency-band EEG signal, also relatively contributes to emotion estimation.

For HRV indexes, it is suggested that LF, HF, and LF/HF, which were the indexes acquired from the frequency-domain analysis of HRV with long time intervals, contribute to emotion estimation. Since the analysis section was long, it was highly possible that these indexes may reflect the state at rest more than the state at which the emotional stimuli were presented, which make it difficult for those indexes to contribute to emotion estimation. However, our results were contrary to this presumption. Though the HRV indexes are related to sympathetic and parasympathetic nervous systems, there is a time lag between the time when the stimulus is presented and the time when the HRV index reflects the influence by the stimulus. From this point of view, the analysis interval is often set to 24 h or 5 min in order to calculate reliable HRV indexes that fully reflects the effects of sympathetic nerves and parasympathetic nerves. In this study, it is considered that LF, HF, and LF/HF have a relatively long analysis interval, which enhances the reliability of the indexes and, as a result, contributes to emotion estimation. Next, RMSSD, which has a high degree of contribution to emotion estimation, is a time-domain HRV index that has been reported to have the same reliability even in a short analysis section like five minutes [42] and is used for monitoring the athlete’s condition. In addition, this index is suggested to have a high reliability for emotion estimation, despite the short analysis interval [43].

Among the top 10 features of the emotion classification for low and high arousal (Figure 8), the number of EEG indexes is one and the number of HRV indexes is nine. Since EEG indexes are used to measure concentration and arousal, we expected that the number of EEG indexes would be more than that of HRV indexes. However, the results differed from our expectations. Related studies suggested that concentration and arousal can be estimated by HRV [44,45,46], which resembles our result. Thus, we suggest that HRV is more useful than EEG for estimating low and high arousal.

On the other hand, among the top 10 features of the emotion classification low and high valence (Figure 9), the number of EEG indexes is four and the number of HRV indexes is six. Since HRV indexes are generally used to measure relaxation and stress, we expected that the number of HRV indexes would be more than that of EEG indexes. The result is the same as expected. In addition, some EEG indexes almost have the same feature importance as HRV indexes. Hence, there is a potential that the EEG indexes can also be used to estimate valence in addition to the HRV indexes. Related studies suggested that EEG is strongly correlated with valence and is useful for estimating low and high valence [3], which resembles our result.

In addition, there is a potential that the EEG indexes calculated from the AF3 node may be replaced by HRV indexes in the classification of low and high valence. This suggests that only HRV may be enough to estimate emotion even without EEG, which contributes to the simplification of emotion estimation technology.

## 5. Accuracy Verification and Discussion

In order to clarify the usefulness of applying our proposed feature selection method, multimodal physiological indexes for the emotion classification model were constructed in this study; accuracy verification was performed using a combination of several features. For accuracy verification, we employed several cross-validation methods with an emotion estimation model constructed using a deep learning algorithm.

### 5.1. Combination of Features

Table 3 shows 21 groups of feature combinations from EEG and/or HRV indexes. Three criteria to group feature combinations were applied as described below.

1. The features were selected based on types of physiological indexes and calculation methods (Groups #1 to #5).
2. All features employed in this research were selected (Group #6).
3. The features were selected based on our proposed four feature selection methods (i.e., ensemble of the four feature selection methods, correlation ratio, mutual information, importance of random forest, and SVM L1 regularization weight) and the three emotion classification models (i.e., “HAHV, HALV, LALV, and LAHV”, “Low/High Arousal”, and “Low/High Valence”) (Groups #7 to #21).

Based on the above criteria, the selected features and the selection method of each group are described as follows:

1. EEG group (#1) consists of all 11 EEG indexes employed in this study.
2. MA15 EEG group (#2) consists of 15-window-sized moving averages of all 11 EEG indexes.
3. TD HRV group (#3) consists of indexes calculated by all 11 HRV indexes calculated by time-domain analysis.
4. FD HRV group (#4) consists of indexes calculated by all 3 HRV indexes calculated by frequency-domain analysis.
5. TD HRV + FD HRV group (#5) consists of the combination of indexes from TD HRV (#3) and FD HRV (#4) groups.
6. ALL group (#6) consists of indexes that combines the indexes from EEG (#1), MA15 EEG (#2), TD HRV (#3), and FD HRV (#4).
7. ENSEMBLE (HAHV, HALV, LALV, and LAHV) group (#7) consists of the top 10 indexes that contribute to emotion estimation in the four-class emotion classification of HAHV, HALV, LALV, and LAHV.
8. ENSEMBLE (Low/High Arousal) group (#8) consists of the top 10 indexes that have the largest contribution of emotion estimation in the binary emotion classification into Low Arousal and High Arousal.
9. ENSEMBLE (Low/High Valence) group (#9) consists of the top 10 indexes that have the largest contribution in the binary emotion classification into Low Valence and High Valence.
10. CR (HAHV, HALV, LALV, and LAHV) group (#10) consists of the top 10 indexes from the correlation ratio as feature selection method that contribute to emotion estimation in the four-class emotion classification of HAHV, HALV, LALV, and LAHV.
11. CR (Low/High Arousal) group (#11) consists of the top 10 indexes from the correlation ratio as the feature selection method that contribute to emotion estimation in the four-class emotion classification of Low Arousal and High Arousal.
12. CR (Low/High Valence) group (#12) consists of the top 10 indexes from the correlation ratio as feature selection method that contribute to emotion estimation in the four-class emotion classification of Low Valence and High Valence.
13. MI (HAHV, HALV, LALV, and LAHV) group (#13) consists of the top 10 indexes from the mutual information as feature selection method that contribute to emotion estimation in the four-class emotion classification of HAHV, HALV, LALV, and LAHV.
14. MI (Low/High Arousal) group (#14) consists of the top 10 indexes from the mutual information as the feature selection method that contribute to emotion estimation in the four-class emotion classification of Low Arousal and High Arousal.
15. MI (Low/High Valence) group (#15) consists of the top 10 indexes from the mutual information as the feature selection method that contribute to emotion estimation in the four-class emotion classification of Low Valence and High Valence.
16. RF (HAHV, HALV, LALV, and LAHV) group (#16) consists of the top 10 indexes from the mutual information as the feature selection method that contribute to emotion estimation in the four-class emotion classification of HAHV, HALV, LALV, and LAHV.
17. RF (Low/High Arousal) group (#17) consists of the top 10 indexes from the importance of random forest as the feature selection method that contribute to emotion estimation in the four-class emotion classification of Low Arousal and High Arousal.
18. RF (Low/High Valence) group (#18) consists of the top 10 indexes from the importance of random forest as the feature selection method that contribute to emotion estimation in the four-class emotion classification of Low Valence and High Valence.
19. SVM L1 (HAHV, HALV, LALV, and LAHV) group (#19) consists of the top 10 indexes from the SVM L1 regularization weight as the feature selection method that contribute to emotion estimation in the four-class emotion classification of HAHV, HALV, LALV, and LAHV.
20. SVM L1 (Low/High Arousal) group (#20) consists of the top 10 indexes from the SVM L1 regularization weight as the feature selection method that contribute to emotion estimation in the four-class emotion classification of Low Arousal and High Arousal.
21. SVM L1 (Low/High Valence) group (#21) consists of the top 10 indexes from the SVM L1 regularization weight rest as the feature selection method that contribute to emotion estimation in the four-class emotion classification of Low Valence and High Valence.

### 5.2. Cross Validation

We selected the stratified K-fold (SKF) method to perform cross validation. It is a cross-validation method in which the ratio of the amount of data of each type of objective variable is equal when dividing training data and test data into K pieces [47]. In general, if there is a bias in the ratio of the amount of correct data for each of the training and test data, the amount of class 1 data will increase in the training data, while the number of class 1 data will decrease in the test data. The amount of data may result in unfair accuracy verification. Therefore, SKF was used to reduce these problems. For this cross-validation method, we set k to 10 which was used to divide data into 10 folds after merging the data of all participants. By using this method, the accuracy of emotion estimation model in which the data of all participants were included in the training data was calculated.

### 5.3. Accuracy Verification Indexes

We used Macro F1 as the accuracy verification index. Macro F1 is an extension of F1-score, which is an accuracy verification index used in binary classification to multi-label classification. The calculation method of F1-score is expressed by Equations (6)–(8).

(6) $$\mathrm{precision}=\frac{\mathrm{TP}}{\mathrm{TP}+\mathrm{FP}}$$

(7) $$\mathrm{recall}=\frac{\mathrm{TP}}{\mathrm{TP}+\mathrm{FN}}$$

(8) $$F1-\mathrm{score}=\frac{2\times \mathrm{recall}\times \mathrm{precision}}{\mathrm{recall}+\mathrm{precision}}$$

In the binary classification of positive and negative examples, the denotations of the variables in the equation is described as follows: TP denotes the amount of data for which the predicted value is a positive example and the prediction is correct; TN denotes the amount of data for which the predicted value is a negative example and the predicted value is correct; FP denotes the amount of data for which the predicted value is a positive example and the prediction is incorrect; FN denotes the amount of data for which the prediction is incorrect using the predicted value as a negative example; precision is an accuracy index that is emphasized when you want to reduce false positives; and recall is an accuracy index that is important when you want to avoid overlooking positive examples; F1-score is a balanced index by taking the harmonic mean of these accuracy indexes. Macro F1 is an accuracy index that calculates the above F1-score for each type of objective variable and their average value [47].

To construct a deep learning model, we used the same structure of the model constructed by Urabe et al. [26]: Intermediate layer: 256-dimensional three layers; intermediate layer activation function: ReLU; output layer activation function: Softmax; optimization algorithm: Stochastic Gradient Descent (SGD); and Dropout: 0.0.

### 5.4. Accuracy Verification Results

**Figure 10:**
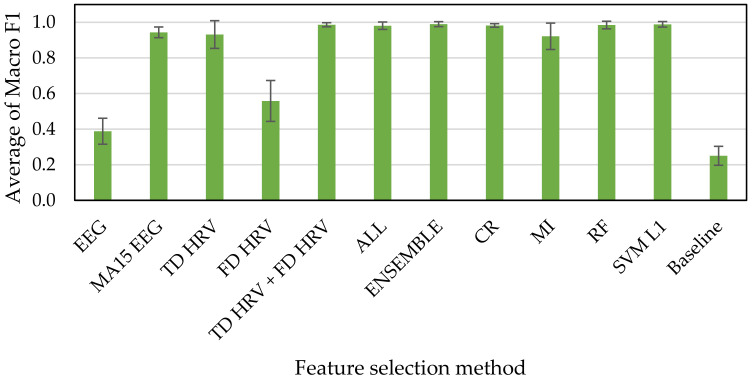
HAHV, HALV, LALV, LAHV (4 classes) emotion classification accuracies.

**Figure 11:**
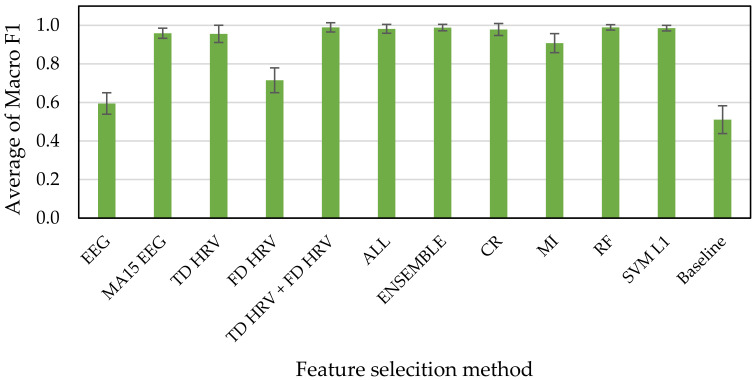
Low Arousal, High Arousal (2 classes) emotion classification accuracies.

**Figure 12:**
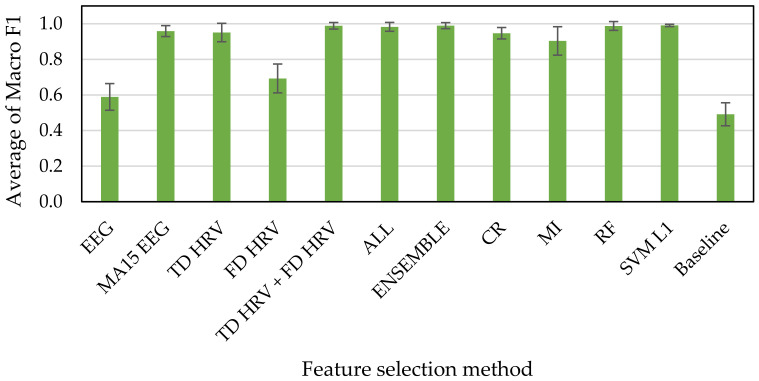
Low Arousal, High Valence (2 classes) emotion classification accuracies.

Figure 10, Figure 11 and Figure 12 show the results of accuracy verification from the cross validations, the accuracy evaluation indexes, and the deep learning. The baseline accuracy of the classification model that returns a random prediction without learning is used as the baseline for accuracy comparison.

First, we compared the accuracies of every methods for feature selection with that of baseline. The results of the accuracy verification using Macro F1 scores as index showed that the accuracies range from 39% to 99% exceeding the baseline of 25% for the HAHV, HALV, LALV, and LAHV model (Figure 10), 59% to 99% exceeding the baseline of 51% for the Low and High Arousal classification model (Figure 11), and 59% to 99% exceeding the baseline of 49% for the Low and High Valence classification model (Figure 12).

Next, we compared the accuracies of our proposed methods (i.e., ENSEMBLE, CR, MI, RF, and SVM L1 groups) with that of all features (i.e., ALL group). The results show that all of them have high accuracies, ranging from 90% to 99% for all three classification models (i.e., “HAHV, HALV, LALV, and LAHV”, “Low Arousal and High Arousal”, and “Low Arousal and High Valence” models). These results indicate that even if all features were not used, the accuracy can reach 99%, which indicates the effectiveness of our proposed feature selection methods used in this study. Since a larger number of features makes the training time take longer in machine learning, we suggest reducing the time spent on training by reducing the number of features while the accuracy is still maintained.

Finally, we compared the accuracies of our proposed methods (i.e., ENSEMBLE, CR, MI, RF, and SVM L1 groups) with those of methods based on the types of physiological indexes and calculation methods (i.e., EEG, MA15 EEG, TD HRV, FD HRV, TD HRV + FD HRV groups). The results show that the accuracies of EEG and FD HRV groups are much lower than those of our proposed methods. In addition, even the accuracies of MA15 EEG, TD HRV, and TD HRV + FD HRV groups are almost the same as those of our proposed methods, they tended to have large variabilities indicating by the standard deviation illustrating as the error bars, especially for the “HAHV, HALV, LALV, and LAHV” emotion classification model. Therefore, we suggested that it is more effective to apply feature selection techniques for constructing the emotion classification model.

Even though the feature selection methods based on the types of physiological indexes and calculation methods are less reliable than our proposed method. By comparing the accuracies between EEG and MA15 EEG groups among the three emotion classification models, the result shows that the accuracy of EEG MA15 group is 56% increased at maximum from that of the EEG group. This result suggests that the accuracy was improved more than double when the moving average is applied to the indexes, suggesting the effectiveness of the moving average.

## 6. Discussion

In this research, we employed feature selection to improve the accuracy of an emotion classification model. We proposed four feature selection methods: correlation ratio (CR), mutual information (MI), importance of random forest (RF), and weight of SVM L1 regularization (SVM L1). In addition, we proposed the feature selection ensemble that combines the results from those four feature selection methods. Based on these, we obtained important features that were later used for model construction.

For accuracy verification, we constructed several emotion classification models using the feature combinations (Table 3) selected based on several criteria, including our proposed methods. As a result, we obtained the following findings:

- A model with high accuracy can be achieved even without using all features from physiological signals, suggesting that the accuracy is not always improved by combining large number of multimodal physiological indexes.
- The model using features only from specific physiological indexes, such as EEG or HRV, may produce a high accuracy; however, the variability tends to be large.
- The accuracy can be improved by applying the moving average to the normal values of physiological indexes.

Based on the above findings, we clarified that our proposed methods by using feature selection successfully improved the accuracy of the emotion classification model. In addition, as our proposed methods selected only top 10 important features, the training time for machine-learning based model can be reduced. Our research results contribute to the improvement of an emotion classification model with a higher accuracy, less cost, and that is less time consuming, which has potential to be further applied to various areas of applications.

This study has some limitations. First, we only collected the physiological signal data from a small number of participants that was unbalanced between males and females. In addition, we employed only eight music pieces (two for each of the four emotions in the Arousal–Valence space model), which might not be enough to fulfil the variety of music preferences among the participants and may result in not completely evoking target emotions. Therefore, we need to employ more music pieces and collect data from a larger number of participants in order to increase the reliability of our experimental results. In addition, as this is our first trial, we selected only four feature selection methods that can easily observe feature importance, and only one method for the cross validation in accuracy verification. Other feature selections, such as neural network, as well as cross validation methods, such as leave-one-subject-out (LOSO), should also be included and compared with our current proposed method in order to increase even higher accuracy for emotion classification model.

To construct the model for accuracy verification, we employed a deep learning algorithm which has shown great advantages in many research fields in recent year [48]. It has been proved to outperform the anomaly detection of medical images at a large scale [49]. Several techniques can be applied to improve the accuracy of deep learning models, such as data augmentation [50,51], the improvement of the capability to handle unseen data [52,53], and the adjustment of structures and parameters to train deep learning models which is typically an important process in every machine learning algorithm. Therefore, we will employ these promising techniques to increase the accuracy of our emotion classification model in order to enable the generalization capability.

## 7. Conclusions

Using an inexpensive and simple EEG and PPG sensors, we extracted and selected the features of the EEG and HRV indexes for the purpose of improving the accuracy of emotion estimation. We proposed feature selection as a method to improve the model accuracy. In order to verify the effectiveness of feature selection, several feature combinations of EEG and/or HRV indexes selected based on our criteria including our proposed feature selection have been used to construct emotion estimation models by deep learning algorithm. The accuracy verification was then performed with the stratified K-fold (SKF) cross-validation method. As a result, we suggest that it is possible to construct an emotion classification model using only a small number of features from physiological indexes. In addition, it was shown that the time spent on training can be shortened by reducing the features while maintaining an accuracy of 98% via appropriate feature selection methods.

For our future work, we will continue to improve our proposed feature selection method, as well as the model accuracy verification, which will enable the generalization of our emotion classification model.

## Figures and Tables

**Table 1:** Electroencephalogram (EEG) indexes used in this study.

EEG Index/Band	Frequency Band (Hz)	Interpretation
δ	1–3	Deepest sleep without dreams, unconscious, non-REM sleep, cognitive task by frontal lobe
θ	4–7	Intuitive, creative, dream, recall, fantasy, imaginary, REM sleep
α ^1^	8–12	Relaxed but not sleepy, tranquil, conscious
β ^2^	13–30	Stress, wide awake, excited, conscious
γ ^3^	31–50	Cognition, motor function, higher mental activity
Low α	8–9	Relaxed, peaceful, conscious
High α	10–12	Relaxed but focused
Low β	13–17	Thinking, accidents and environmental awareness, relaxed yet focused, integrated
High β	18–30	Alert, upset, agitation
Low γ	31–40	Memory, higher mental activity
Mid γ	41–50	Visual information processing
MA15 × ^4^ where x = {θ, δ, α, β, γ, Low α, High α, Low β, High β, Low γ, Mid γ}	Note ^5^	Note ^5^

^1^ α is calculated from Low α + High α. ^2^ β is calculated from Low β + High β. ^3^ γ is calculated from Low γ + Mid γ. ^4^ Moving average of index x with window size of 15. ^5^ The frequency band and interpretation are corresponding to each EEG index/band x.

**Table 2:** Heart Rate Variability (HRV) indexes used in this study.

HRV Index	Definition	Interpretation
Inter-beat Interval (IBI)	Time interval between adjacent heartbeats	Sympathetic and parasympathetic nerves
Heart Rate (HR)	Number of beats per minute	Tension, Calm
pNNx ^1^ where x = {10, 20, 30, 40, 50}	Percentage of adjacent IBIs with absolute values greater than x ms	Parasympathetic nerve
SDNN ^1^	Standard deviation of IBI	Sympathetic and parasympathetic nerves
RMSSD ^1^	Root mean square of IBI difference	Parasympathetic nerve
SDNN/RMSSD ^1^	Ratio of SDNN by RMSSD	Sympathetic nerve
CVNN ^1^	Coefficient of variation of IBI	Sympathetic and parasympathetic nerves
LF ^2^	Frequency-domain analysis of IBI power value of 0.04–0.15 Hz	Sympathetic and parasympathetic nerves
HF ^2^	Frequency-domain analysis of IBI power value of 0.15–0.40 Hz	Parasympathetic nerve
LF/HF ^2^	LF/HF	Sympathetic nerve

^1^ Every time an IBI value is acquired, the value is calculated with the interval of 30. ^2^ Every time an IBI value is acquired, the value is calculated with an interval of 200 s.

**Table 3:** Groups of feature combinations from EEG and/or HRV indexes.

Group No.	Group Name	Feature Combination
#1	EEG	θ, δ, Low α, High α, Low β, High β, Low γ, Mid γ, α, β, γ
#2	MA15 EEG	MA15 θ, MA15 δ, MA15 Low α, MA15 High α, MA15 Low β, MA15 High β, MA15 Low γ, MA15 Mid γ, MA15 α, MA15 β, MA15 γ
#3	TD HRV	IBI, HR, CVNN, SDNN, RMSSD, SDNN/RMSSD, pNN10, pNN20, pNN30, pNN40, pNN50
#4	FD HRV	LF, HF, LF/HF
#5	TD HRV + FD HRV	IBI, HR, CVNN, SDNN, RMSSD, SDNN/RMSSD, pNN10, pNN20, pNN30, pNN40, pNN50, LF, HF, LF/HF
#6	ALL	θ, δ, Low α, High α, Low β, High β, Low γ, Mid γ, α, β, γ, MA15 θ, MA15 δ, MA15 Low α, MA15 High α, MA15 Low β, MA15 High β, MA15 Low γ, MA15 Mid γ, MA15 α, MA15 β, MA15 γ, IBI, HR, CVNN, SDNN, RMSSD, SDNN/RMSSD, pNN10, pNN20, pNN30, pNN40, pNN50, LF, HF, LF/HF
#7	ENSEMBLE (HAHV, HALV, LALV, LAHV)	LF, HF, LF/HF, RMSSD, SDNN, MA15 Mid γ, CVNN, pNN30, MA15 δ, pNN40
#8	ENSEMBLE (Low/High Arousal)	RMSSD, SDNN/RMSSD, LF, LF/HF, HF, pNN30, pNN40, CVNN, SDNN, MA15 δ
#9	ENSEMBLE (Low/High Valence)	LF, MA15 Mid γ, HF, RMSSD, MA15 δ, LF/HF, SDNN, MA15 Low γ, MA15 γ, pNN40
#10	CR (HAHV, HALV, LALV, LAHV)	MA15 Mid γ, LF/HF, MA15 γ, MA15 δ, MA15 Low γ, MA15 High β, SDNN/RMSSD, LF, MA15 High α, MA15 α
#11	CR (Low/High Arousal)	SDNN/RMSSD, LF/HF, MA15 δ, RMSSD, pNN10, MA15 Low α, MA15 Mid γ, MA15 Low β, Low α, pNN30
#12	CR (Low/High Valence)	MA15 Mid γ, MA15 γ, MA15 δ, MA15 Low γ, MA15 α, MA15 Low α, MA15 θ, pNN50, MA15 High α, γ
#13	MI (HAHV, HALV, LALV, LAHV)	RMSSD, LF, HF, SDNN, CVNN, LF/HF, β, High β, Mid γ, γ
#14	MI (Low/High Arousal)	RMSSD, LF, HF, SDNN, High α, δ, Low β, θ, β, CVNN
#15	MI (Low/High Valence)	RMSSD, LF, β, SDNN, HF, γ, High β, δ, Low β, CVNN
#16	RF (HAHV, HALV, LALV, LAHV)	LF, HF, LF/HF, RMSSD, CVNN, SDNN/RMSSD, SDNN, MA15 Low γ, MA15 Mid γ, MA15 High β
#17	RF (Low/High Arousal)	LF, LF/HF, HF, RMSSD, SDNN/RMSSD, MA15 High β, CVNN, MA15 δ, MA15 θ, SDNN
#18	RF (Low/High Valence)	LF, HF, LF/HF, RMSSD, MA15 Low γ, CVNN, MA15 High β, MA15 Mid γ, MA15 High α, SDNN/RMSSD
#19	SVM L1 (HAHV, HALV, LALV, LAHV)	SDNN, pNN30, LF, HF, pNN40, pNN20, CVNN, pNN10, pNN50, MA15 Mid γ
#20	SVM L1 (Low/High Arousal)	pNN30, pNN40, MA15 Low β, pNN20, pNN10, RMSSD, MA15 δ, HR, MA15 High β, MA15 Low γ
#21	SVM L1 (Low/High Valence)	LF, HF, MA15 Mid γ, pNN40, MA15 δ, RMSSD, pNN30, pNN20, LF/HF, HR,

## Data Availability

Restrictions apply to the availability of the soundtrack datasets for music and emotion. The data were obtained from the University of Jyväskylä and are available on request.
